# Rare Synaptogenesis-Impairing Mutations in *SLITRK5* Are Associated with Obsessive Compulsive Disorder

**DOI:** 10.1371/journal.pone.0169994

**Published:** 2017-01-13

**Authors:** Minseok Song, Carol A. Mathews, S. Evelyn Stewart, Sergey V. Shmelkov, Jason G. Mezey, Juan L. Rodriguez-Flores, Steven A. Rasmussen, Jennifer C. Britton, Yong-Seok Oh, John T. Walkup, Francis S. Lee, Charles E. Glatt

**Affiliations:** 1 Synaptic Circuit Plasticity Laboratory, Department of Structure & Function of Neural Network, Korea Brain Research Institute, 61 Cheomdan-ro, Dong-gu, Daegu, Korea; 2 Department of Psychiatry, University of California San Francisco, San Francisco, California, United States of America; 3 Department of Psychiatry, Faculty of Medicine, University of British Columbia, Vancouver, Canada; 4 Department of Neuroscience and Physiology, New York University School of Medicine, New York, NY, United States of America; 5 Department of Psychiatry, New York University School of Medicine, New York, NY, United States of America; 6 Department of Biological Statistics and Computational Biology, Cornell University, Ithaca, NY United States of America; 7 Department of Genetic Medicine, Weill Cornell Medical College, NY, NY United States of America; 8 Department of Psychiatry and Human Behavior, Alpert Medical School, Brown University, Providence, RI, United States of America; 9 Department of Psychology, University of Miami, Miami, FL, United States of America; 10 Department of Brain-Cognitive Science, Daegu-Gyeongbuk Institute of Science and Technology (DGIST), Hyeonpung-myeon, Dalseong-gun, Daegu, Republic of Korea; 11 Department of Psychiatry, Weill Cornell Medical College, New York, New York, United States of America; 12 Sackler Institute for Developmental Psychobiology, Weill Cornell Medical College, New York, New York, United States of America; Augusta University, UNITED STATES

## Abstract

Obsessive compulsive disorder (OCD) is substantially heritable, but few molecular genetic risk factors have been identified. Knockout mice lacking SLIT and NTRK-Like Family, Member 5 (SLITRK5) display OCD-like phenotypes including serotonin reuptake inhibitor-sensitive pathologic grooming, and corticostriatal dysfunction. Thus, mutations that impair SLITRK5 function may contribute to the genetic risk for OCD. We re-sequenced the protein-coding sequence of the human SLITRK5 gene (*SLITRK5*) in three hundred and seventy seven OCD subjects and compared rare non-synonymous mutations (RNMs) in that sample with similar mutations in the 1000 Genomes database. We also performed *in silico* assessments and *in vitro* functional synaptogenesis assays on the Slitrk5 mutations identified. We identified four RNM’s among these OCD subjects. There were no significant differences in the prevalence or *in silico* effects of rare non-synonymous mutations in the OCD sample versus controls. Direct functional testing of recombinant SLITRK5 proteins found that all mutations identified in OCD subjects impaired synaptogenic activity whereas none of the pseudo-matched mutations identified in 1000 Genomes controls had significant effects on SLITRK5 function (Fisher’s exact test P = 0.028). These results demonstrate that rare functional mutations in *SLITRK5* contribute to the genetic risk for OCD in human populations. They also highlight the importance of biological characterization of allelic effects in understanding genotype-phenotype relationships as there were no statistical differences in overall prevalence or bioinformatically predicted effects of OCD case versus control mutations. Finally, these results converge with others to highlight the role of aberrant synaptic function in corticostriatal neurons in the pathophysiology of OCD.

## Introduction

Obsessive compulsive disorder (OCD) is a neuropsychiatric disorder consisting of persistent, intrusive, distressing thoughts and repetitive, compulsive behaviors and mental rituals [1]. Epidemiologic studies have determined that OCD displays a substantial heritable component of risk, however, few specific genetic risk factors have been identified [2–4]. Recent large-scale genome wide association studies (GWAS) of OCD have identified common polymorphisms that are associated with OCD at near genome-wide significance levels [5, 6]. These studies have demonstrated that the genetic architecture of OCD is very complex likely consisting of hundreds to thousands of common polymorphisms each of small effect size. The small effect sizes of these risk alleles prevents their practical usage as clinical biomarkers but OCD GWAS’s have begun to identify biological processes in which the associated polymorphisms are enriched and thus presumably underlie the pathophysiology of OCD.

Recently it has been appreciated that, in addition to common polymorphisms, rare genetic variation can contribute to the risk for neuropsychiatric disorders in human populations [7]. RNM’s have been implicated in genetic risk for both autism and schizophrenia through whole exome sequencing [8, 9]. In these studies, rare variants are enriched in cases versus controls to a degree suggesting that they have large effects on autism risk relative to common polymorphisms. In OCD, targeted re-sequencing of the human gene for the postsynaptic synapse-associated protein 90 (SAP90)/postsynaptic density-95 (PSD95)-associated protein 3 (*SAPAP3*), motivated by an OCD-like phenotype in knockout mice lacking SAPAP3, found an overrepresentation of RNM’s in OCD and/or trichotillomania subjects implicating *SAPAP3* in the genetic risk for OCD [10].

We have identified an OCD-like phenotype in mice lacking expression of SLIT and NTRK-Like Family, Member 5 (SLITRK5) [11]. SLITRK5 knockout mice display a pathologic over-grooming phenotype that is accompanied by disrupted corticostriatal circuit activity. Moreover, pathologic grooming behavior in SLITRK5 knockout mice is normalized by serotonin reuptake inhibitors; the most effective pharmacologic treatments for OCD [12, 13].

The SLITRK’s are a family of transmembrane proteins that have two extracellular leucine rich repeat (LRR) domains which facilitate protein-protein interactions [14]. In particular, postsynaptic SLITRK3 has been shown to facilitate inhibitory synaptogenesis through trans-synaptic interactions with presynaptic protein tyrosine phosphatase delta (PTPδ) [15]. All of the SLITRK family members bind PTPδ, and a recently reported structural study of PTPδ and SLITRK1 supports the notion that other SLITRK isoforms may also play a role in synapse formation via interaction with PTPδ [16]. Slitrk5 expression is enriched in striatal neurons suggesting that altered synaptogenesis due to loss of SLITRK5-PTPδ interactions may provide a mechanism for the selective corticostriatal phenotypes seen in the SLITRK5 knockout mouse: decreased striatal volume, decreased dendritic complexity of striatal neurons, reduced expression of glutamate receptor subunits on striatal neurons, and decreased post-synaptic responses of striatal neurons to stimulation by cortical inputs [11].

Motivated by the phenotypic similarities between SLITRK5 knockout mice and human OCD, we hypothesized that, as with *SAPAP3*, RNM’s in the human SLITRK5 gene (*SLITRK5)* might contribute to the genetic risk for OCD. We re-sequenced the protein coding sequence of *SLITRK5* in human subjects with OCD and compared RNM’s (population prevalence <1%) from that sample with mutations in the 1000 Genomes Database [17]. Although there were no statistical differences in the distribution or bioinformatically predicted functional effects of SLITRK5 mutations from OCD cases versus controls, direct functional testing determined that all of the *SLITRK5* mutations identified in OCD subjects reduced synaptogenesis *in vitro* while none of the most comparable pseudo-matched control mutations from the 1000 Genomes Database we tested had significant effects. These results implicate *SLITRK5* in the population risk for OCD and highlight the role of synaptic function in corticostriatal circuitry in the pathophysiology of OCD.

## Methods

### Samples for re-sequencing

The re-sequencing sample consisted of three hundred and seventy seven OCD subjects (754 chromosomes). All subjects provided written informed consent after receiving a complete description of the study and met DSM-IV-TR criteria for OCD [1]. Details of subject recruitment and ascertainment have been published in other reports (5, 6, 37–39). The ancestral composition of the re-sequencing sample was mixed with major representation of European American (68%) and Hispanic chromosomes (11%).

For control chromosomes we used data from the initial 1092 subject (2184 chromosomes) 1000 Genomes Project sample of un-phenotyped individuals of diverse ancestry (17).

### Re-sequencing

Overlapping amplicons covering the entire protein-coding sequence of human *SLITRK5* were amplified for each subject by polymerase chain reaction (PCR). Sequences for PCR primers are available in supplemental material. After removal of excess primers and free nucleotides samples were sequenced by Sanger sequencing. Mutations were identified by visual inspection of aligned sequencing results using Sequencher software (Gene Codes). All putative mutations were confirmed by sequencing of the reverse strand from independent PCR reactions.

### *In silico* annotation of SLITRK5 mutations

Bioinformatic assessment of the deleteriousness of mutations in *SLITRK5* was performed using Combined Annotation Dependent Depletion (CADD; http://cadd.gs.washington.edu), which combines information from multiple annotations to quantitatively prioritize functional variants (20). Functional annotation was based on Human Genome Assembly build 65 and GRCh37 human reference genome.

### Mice

All animal procedures were approved by the Institutional Animal Care and Use Committees of Weill Cornell Medical College and were conducted in accordance with the National Institutes of Health Guide for the Care and Use of Laboratory Animals. Pregnant mice around E16 (from Charles River) were maintained in a SPF barrier facility under a 11:13-h light:dark cycle (lights on, 0700 to 1800) at temperatures of 21 to 24°C. Mice were housed in individually ventilated cages on corncob bedding with ad-libitum access to food (Harlan Global Diet Low Fat Irradiated) and water. Mice were sacrificed by decapitation after being anesthetized by CO2 in accordance with the guidelines of the U.S. Animal Welfare Act. All efforts were made to minimize animal suffering, to reduce the number of animals used.

### Functional Assays

Detailed methods including reagents are available in supplemental material.

#### Synaptogenesis assay

Primary hippocampal mouse neurons were co-cultured with HEK-293 cells that had been individually transfected with plasmids containing human influenza hemagglutinin (HA)-tagged versions of wild type SLITRK5, *SLITRK5* mutants from OCD cases or 1000 Genome controls, and a negative control form of SLITRK5 lacking the extracellular domain (ΔECD). SLITRK5 expressing transfected cells were identified by immunolabeling with anti-HA antibodies. Synaptogenesis by hippocampal neurons onto SLITRK5 expressing HEK-293 cells was quantified by immunolabeling hippocampal axons using anti-Tau antibodies [18] and synapses using anti-Synapsin I antibodies [19]. Synaptogenesis was defined as the total intensity of synapsin I signal in regions positive for both surface HA (labeling transfected SLITRK5-expressing HEK-293 cells) and dephospho-Tau (labeling primary hippocampal axons). Analysis was performed using NIS-Elements (Nikon Instruments Inc., NY, USA), Microsoft Excel and GraphPad Prism 4. Statistical comparisons were made using one-way ANOVA with Dunnett's multiple comparisons test, as indicated in the Figure legends. All data are reported as the mean ± s.d. in at least three independent experiments.

#### Subcellular localization assay

HEK-293 cell cultures were individually transfected with recombinant versions of each mutation introduced into the HA-tagged SLITRK5 peptide. HEK-293 cells were fixed and incubated with anti-HA antibodies without permeabilization to specifically label cell surface expressed recombinant SLITRK5. Surface HA-tagged SLITRK5 was visualized by immunolabeling with Alexa-488 dye-conjugated anti-mouse antibodies. Cells were then permeabilized with 0.2% Triton X-100-containing PBS, and internally expressed SLITRK5 alleles were stained with anti-HA antibodies. Internally localized HA-SLITRK5 was visualized with Alexa-568 dye-conjugated anti-mouse antibodies. Cells were examined by fluorescence microscopy and staining intensities of each fluor in individual cells was quantified using NIS-Elements. Values corresponding to surface SLITRK5 (green) were divided by the total fluorescence values (red+green) and normalized to wild type SLITRK5. All data are reported as the mean ± s.d. in at least three independent experiments.

#### Soluble PTPδ-Fc protein and binding assays

Based on previously described methods [15], a soluble PTPδ ectodomain peptide fused to an Fc immunoglobulin domain epitope tag (PTPδ-Fc) was generated by transfecting HEK-293 cells and then purified from culture media. HEK-293 cells on coverslips were transfected with WT or variant HA-tagged SLITRK5-coding plasmids and incubated with the soluble PTPδ-Fc fusion protein (200nM). Cells were then immunolabeled with anti-IgG and anti-HA antibodies. Binding was measured as the average ratio of bound PTPδ-Fc immunofluorescence to HA-SLITRK5 immunofluorescence normalized to wild type SLITRK5. All data are reported as the mean ± s.d. in at least three independent experiments.

## Results

### Re-sequencing *SLITRK5*

We identified four rare non-synonymous mutations (RNM’s) in 377 OCD subjects (1,1%), non-significantly lower than the prevalence of similar mutations in 1092 unphenotyped controls from the 1000 Genomes Database where there were fifteen (1.4%), Chi-square 0.21, P = 0.65. Each of the OCD-associated mutations was seen in a single chromosome and seven of the control mutations were singletons. Of the remaining control mutations, eight were seen in a single population and one was seen in two of the four populations included in the 1092 subject 1000 Genomes sample (European, EUR; Americas, AMR; African, AFR; Asian, ASN). One of the rare mutations from the 1000 Genomes Database had a population-specific prevalence greater than 1% in the ASN sample (1.6%) and was excluded from further analyses to avoid possible downward biasing of the effects of control RNM’s.

The prevalence of chromosomes containing RNM’s was also non-significantly lower in OCD cases (0.5%; 4 singletons/754 chromosomes) than controls (1.3%; 28 total mutant chromosomes/2184 total chromosomes) for controls Chi-square 2.88, P = 0.09. These results demonstrate that there is no association of OCD with either the number of *SLITRK5* RNM’s or the prevalence of chromosomes containing those mutations.

### Comparison of predicted deleterious effects of *SLITRK5* mutations

We assessed the predicted effects of RNM’s *in silico* using CADD which provides a single C score that reflects a prediction of the deleterious functional effects of specific amino acid substitutions. [20]. There were no significant differences in the raw or scaled C scores between RNM’s found in OCD cases versus 1000 Genomes controls or the subset of pseudo-control mutations used for SLITRK5 functional testing (Table 1). There were no differences in the bioinformatically predicted effects of RNM’s in any of the component annotations including Grantham, SIFT, or Polyphen scores [21–23].

**Table 1:** In Silico effects of rare non-synonymous Pmutations. Avg (s.e.m.), p values based on Student's T test.

	OCD Cases	1000 Genomes	Pseudo-matched controls
CADD score	2.43 (0.31)	2.32 (0.26)	2.72 (0.90)
**P value**		**0.77**	**0.78**
normalized CADD	21.27 (0.90)	19.73 (0.83)	20.33 (2.52)
**P value**		**0.19**	**0.75**

### Functional analysis of *SLITRK5* RNM’s

**Fig 1:**
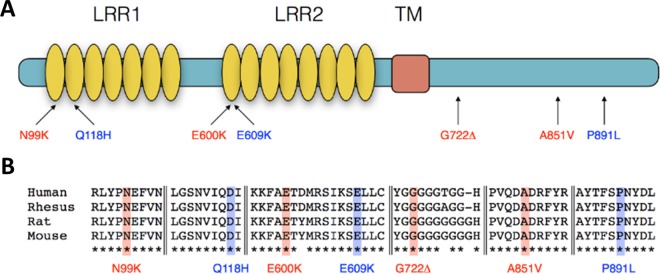
Structure of Slitrk5 and location of rare non-synonymous mutations. (A) Schematic representation of the Slitrk5 protein with extracellular Leucine Rich Repeat (LRR) domains and transmembrane (TM) domain marked. Mutations identified in OCD subjects are labeled in red and pseudo-matched mutations from 1000 Genome Database subjects are labeled in blue. (B) SLITRK5 mutations placed in their primary sequence context. All mutations alter absolutely conserved peptide contexts denoted by asterisks.

For functional testing, we cloned each mutation into an HA epitope tagged fusion protein on the ancestral human SLITRK5 open reading frame background in a mammalian expression vector. We analyzed all OCD mutations and a subset of pseudo-matched mutations from the 1000 Genomes database sample (Fig 1A). Pseudo-matched controls were selected as the mutation nearest to an OCD mutation. For the three amino acid substitution OCD RNM’s there were pseudo-controls in the same functional domain; for the amino acid deletion mutant we could not identify an appropriate pseudo-matched control (Fig 1A). All of the OCD mutations and pseudo-matched controls occur in residues that are absolutely conserved from mouse to human and occur in very highly conserved contexts (Fig 1B). For two of the three pseudo-matched pairs the chemical categories of both the ancestral and mutant amino acids were also matched, for the third pair, the OCD mutation (N99K) is an acidic polar amino to basic amino acid substitution and the pseudo control (Q118H) is an acidic to basic substitution. Average normalized CADD score for OCD mutations (21.27± 0.9) and pseudo-matched controls (20.33 ± 2.52) were statistically similar (P = 0.75; Table 1).

### Synaptogenesis

**Fig 2:**
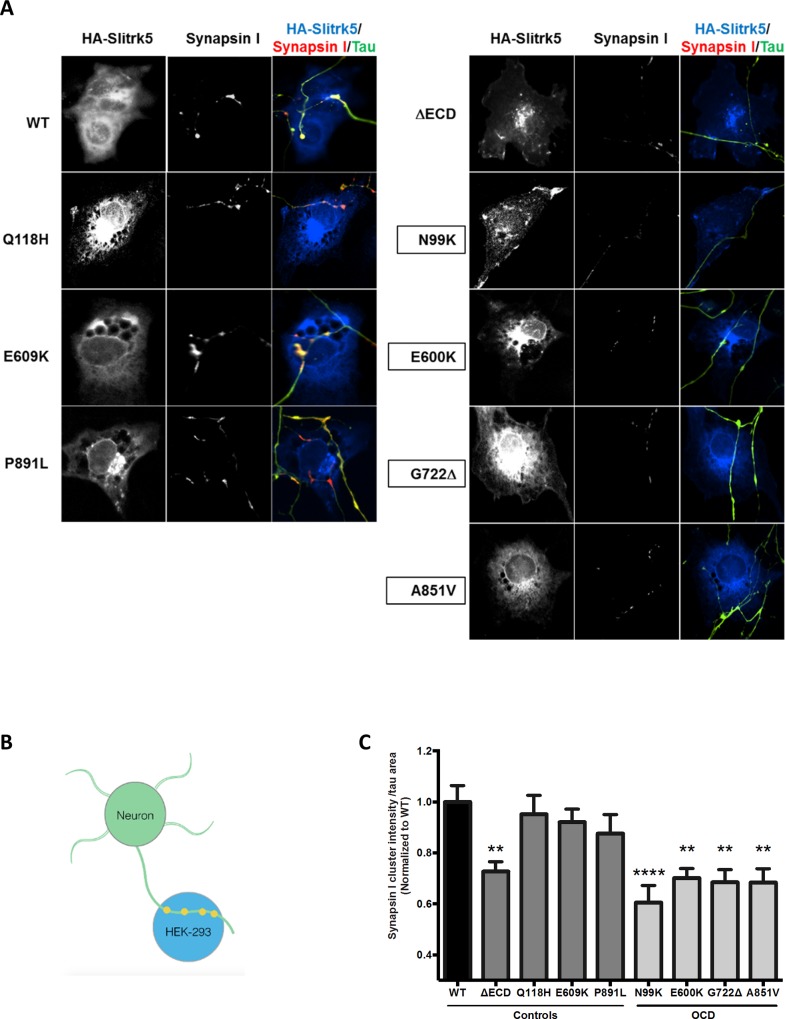
Synapse formation is altered by OCD-associated *SLITRK5* mutations. (A) Representative images from synapse formation assays for control or OCD mutants of Slitrk5. Axonal processes of primary neurons were immunolabeled for the neuronal marker Tau (green), and HEK-293 cells were transfected with and immunostained for HA-tagged recombinant SLITRK5 proteins (blue). Synapsin I immunostaining (red) only occurs where a synapse is formed and the overlap with Slitrk5 appears yellow. Synapse formation is measured as the ratio of immunoreactivity for the synapse marker Synapsin I to the axonal marker Tau. (B) Synapse formation assay. Primary neurons (green) were co-cultured with HEK-293 cells that were transfected with various alleles of Slitrk5 (blue). Synaptogenesis is measured by expression of the synapse-specific marker Synapsin I (orange). (C) All OCD-associated and no control mutants caused reduced synaptogenesis. Results are normalized to the ancestral wild type (WT) human allele. ΔECD is a deletion mutant negative control SLITRK5 lacking the extracellular domain used as a negative control. (25–30 cells were analyzed per condition per experiment. **: P<0.01, ****: P<0.001 compared to WT).

The SLITRK’s are postsynaptic trans-membrane proteins that promote synaptogenesis in co-culture systems through interaction of their leucine rich repeat (LRR) domains [24, 25]. We therefore performed co-culture assays in which non-synaptogenic HEK-293 cells are transfected with recombinant HA-SLITRK5 which then acts as bait attracting synapse formation by primary hippocampal neurons (Fig 2B). Synaptogenesis was measured through immunolabeling and quantifying expression of the synaptic marker, Synapsin I, in Tau-expressing axons overlaying HA-SLITRK5 positive HEK-293 cells (Fig 2A and 2C).

We found that all SLITRK5 alleles containing mutations identified in OCD subjects significantly impaired synapse formation relative to ancestral SLITRK5 when transfected into HEK-293 cells whereas none of the pseudo-matched controls did (Fig 2A and 2C) significantly implicating functional mutations in *SLITRK5* in genetic risk for OCD (Fisher’s exact test P = 0.028).

### Surface expression and TrkB binding

**Fig 3:**
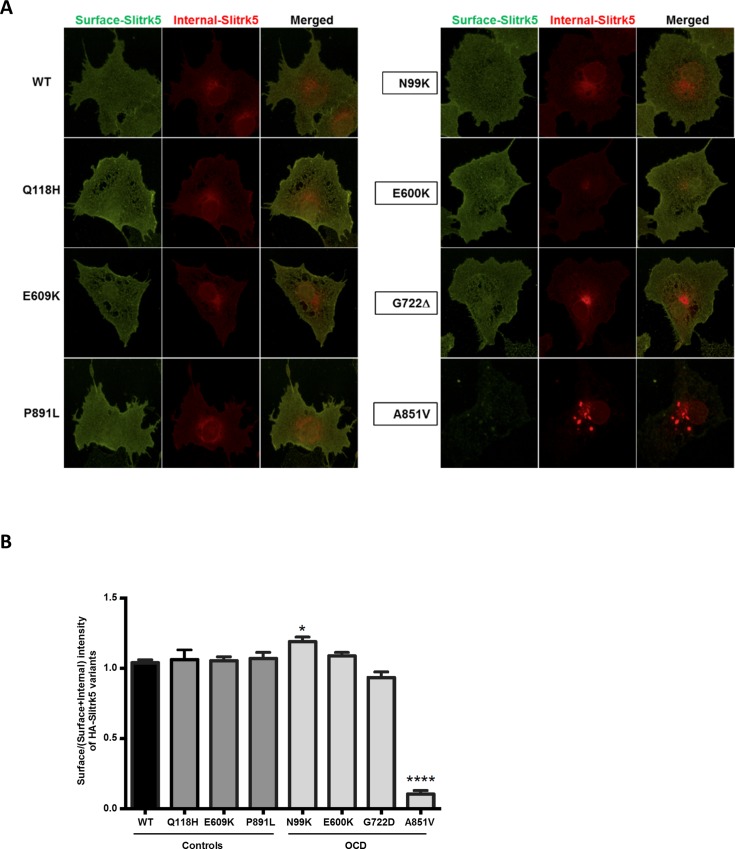
OCD-associated mutation A851V prevents surface expression of SLITRK5. (A) Representative images of immunolabeled surface (green) and cytoplasmic (red) SLITRK5 in control RNM and OCD RNM transfected cells. (B) Surface localization of mutant SLITRK5. Fraction of surface expression measured as SLITRK5 immunolabeling of intact cells divided by the sum of intact and permeabilized labeling. (20–30 cells were analyzed per condition per experiment. *P<0.05, ****P< 0.0001 compared to WT).

To refine the mechanism by which the OCD-associated *SLITRK5* mutations impaired synaptogenesis, we first tested if mutant HA-SLITRK5 alleles were expressed properly at the cell surface. HEK-293 cells were transfected with OCD or control HA-SLITRK5 alleles and surface expression was assessed by immunolabeling HA-SLITRK5 in intact cells followed by immunolabeling of HA-SLITRK5 in the same cells treated with 0.2% Triton X-100 to permeabilize plasma membranes (Fig 3A and 3B). One of the *SLITRK5* mutations identified in an OCD case (A851V) displayed significantly reduced surface localization (0.18 ± 0.03; P<0.0001) while all other mutants, including the pseudo-matched control (P891L) were expressed at the cell surface similarly to ancestral HA-SLITRK5 (Fig 3A and 3B).

Previously, we have reported that *SLITRK5* interacts with and regulates TrkB receptor function. We next considered whether *SLITRK5* RNM’s exhibited deficient TrkB binding. To test this hypothesis, we carried out co-immunoprecipitation experiments using HEK-293 cells transfected with FLAG-tagged TrkB and HA-tagged *SLITRK5* RNM’s. We found that only one OCD-associated mutants (A851V) failed to co-precipitate with TrkB, probably due to altered subcellular localization, whereas the other OCD RNM’s as well as control mutants exhibited intact TrkB binding under resting condition (S1 Fig).

### Protein tyrosine phosphatase delta (PTPδ) binding

**Fig 4:**
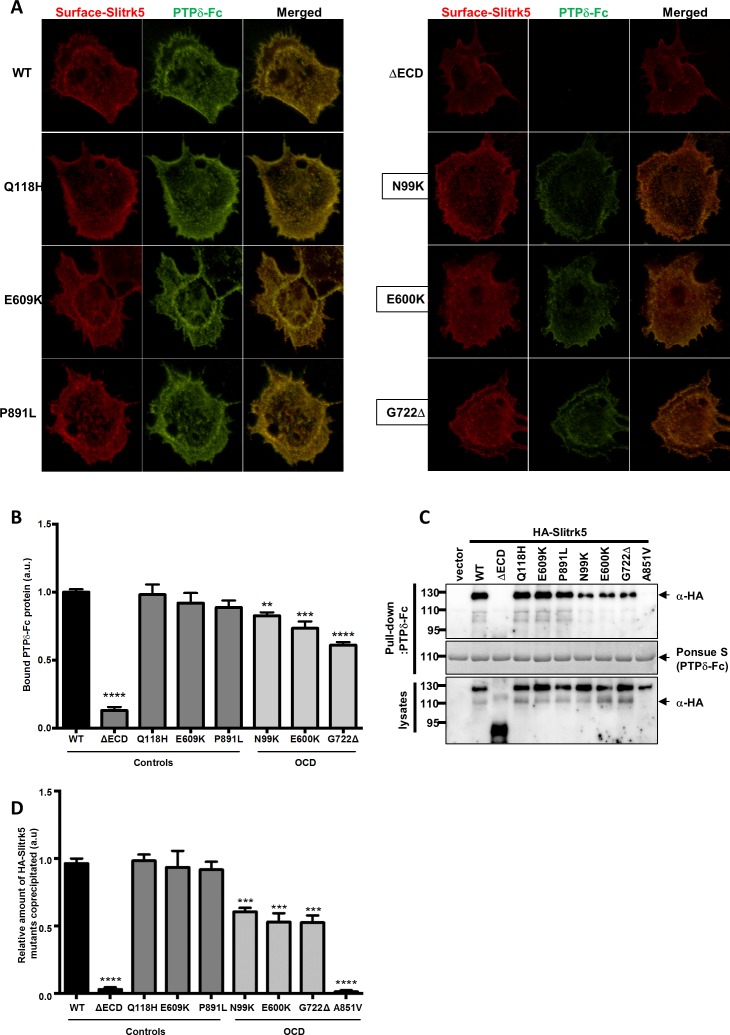
PTPδ-Fc binding is impaired by OCD-associated SLITRK5 mutations. (A) Representative images of HA-SLITRK5 (red) and PTPδ-Fc (green) immuno-fluorescence for control and OCD RNM. HEK-293 cells were transfected with HA-tagged SLITRK5 mutants and incubated with a PTPδ ectodomain-Fc fragment fusion protein. Areas of low SLITRK5-PTPδ binding (OCD mutants) appear orange, higher levels of binding (control mutants) appear yellow. (B) PTPδ binding to Slitrk5 alleles. All four mutations from OCD subjects (data for A851V not shown) displayed significantly reduced PTPδ whereas none of the control mutations did. ΔECD is a negative control SLITRK5 mutant lacking an extracellular domain. 25–30 cells were analyzed per condition per experiment. (C, D) Co-immunoprecipitation of PTPδ to Slitrk5 alleles. All four mutations from OCD subjects displayed significantly reduced co-precipitation with PTPδ, whereas none of the control mutations did. **P<0.01, ***P<0.001, ****P<0.0001

PTPδ is a transmembrane domain protein that binds to members of the SLITRK family. Transsynaptic PTPδ-SLITRK3 interactions have been shown to facilitate formation of inhibitory synapses [15]. We have recently shown that PTPδ competes with TrkB, the prototypic BDNF receptor, for binding to SLITRK5. In the absence of BDNF, SLITRK5 forms transsynaptic bonds with PTPδ whereas in the presence of BDNF SLITRK5 interacts in cis with TrkB [26]. We therefore tested if the remaining mutations, which occur in LRR domains that facilitate protein-protein interactions, alter synaptogenesis by impairing SLITRK5-PTPδ interactions. We incubated soluble fusion protein comprised of the PTPδ ectodomain and the human immunoglobulin Fc fragment with HEK-293 cells transfected with surface expressed mutant HA-SLITRK5 alleles and immunolabeled the fusion proteins through their Fc- and HA- epitope tags (Fig 4A and 4B) [15]. All of the remaining, surface-expressed, OCD mutations displayed reduced binding of PTPδ-Fc (Fig 4B) providing a mechanistic explanation for the reduced synaptogenesis seen in the co-culture studies.

In parallel with this imaging study, we performed biochemical study to assess physical association of PTPδ-Fc with SLITRK5 mutations. After transfection of HEK-293 cells with the control and OCD-associated SLITRK5 mutations, their interaction with purified PTPδ-Fc was examined by co-precipitation and immunoblot analyses. These studies demonstrated that the OCD-associated SLITRK5 mutations exhibited reduced interaction with PTPδ-Fc compared to control mutations. Especially, A851V mutant that does not localized to the plasma membrane showed complete loss of PTPδ-Fc binding (Fig 4C and 4D).

## Discussion

We have re-sequenced *SLITRK5* to identify RNM’s in OCD patients. When comparing the prevalence of OCD RNM’s with controls from the 1000 Genomes Project, there were no significant differences either in the number of distinct RNM’s or their prevalence. Bioinformatic analysis did not predict that RNM’s from OCD cases were more deleterious than those from controls. Direct testing of the synaptogenic activity of recombinant SLITRK5 and mutant alleles found that all of the RNM’s from OCD subjects impaired synaptogenesis whereas none of the control RNM’s did. These results demonstrate that functionally deleterious mutations in *SLITRK5* are significantly associated with OCD. Our use of controls from the 1000 Genomes Database can be critiqued because participants are not true controls as they are not screened to rule out OCD or other disorders however such a large dataset provides more information on rare mutations than would be possible with screened controls. Moreover, any biasing effects of individuals with OCD in the 1000 Genomes Database sample would tend to include potential OCD-associated functional mutations in our control sample and none of the 1000 Genome RNM’s affected synaptogenesis. Similarly, our functional testing of only a subset of 1000 Genomes RNM’s leaves open the possibility that RNM’s not tested may alter synaptogenesis however our focus on the most similar, closest matched pseudo-control RNM’s was intended to test the RNM’s most likely to affect Slitrk5 function in similar ways to the OCD RNM’s and can therefore be considered conservative. Finally, the ethnic composition of our OCD cases is not identical to the populations represented in the 1000 Genomes Database and differences in the prevalence of rare mutations across populations has been identified as a source of artifactual associations [27]. The issue is minimized in this study because the basis of our association is not based on a relative over-abundance of RNM’s in cases versus controls rather on the fact that those RNM’s present in OCD subjects biologically affect synaptogenesis and those in controls do not.

These studies emphasize the importance of integrating biological approaches into human behavioral genetic studies. In the case of common polymorphisms, biological characterization of human variants using *in vivo* model systems can enhance the reliability of genetic association studies of behavioral domains and facilitate exploratory hypothesis generation [28–30]. In this report we have demonstrated that direct biological characterization of RNM’s can be essential in dissociating pathology-related from control variation in instances when the case-control distribution and *in silico* prediction of functional effects of RNM’s do not identify statistical associations. Moreover, association based on impaired biological function reduces the risk of common confounding factors such as ethnic stratification that can be a particular problem in association analyses of rare population-specific mutations [27] because causality is inferred based on the effects of pathology-associated mutations as opposed to their simple existence.

**Fig 5:**
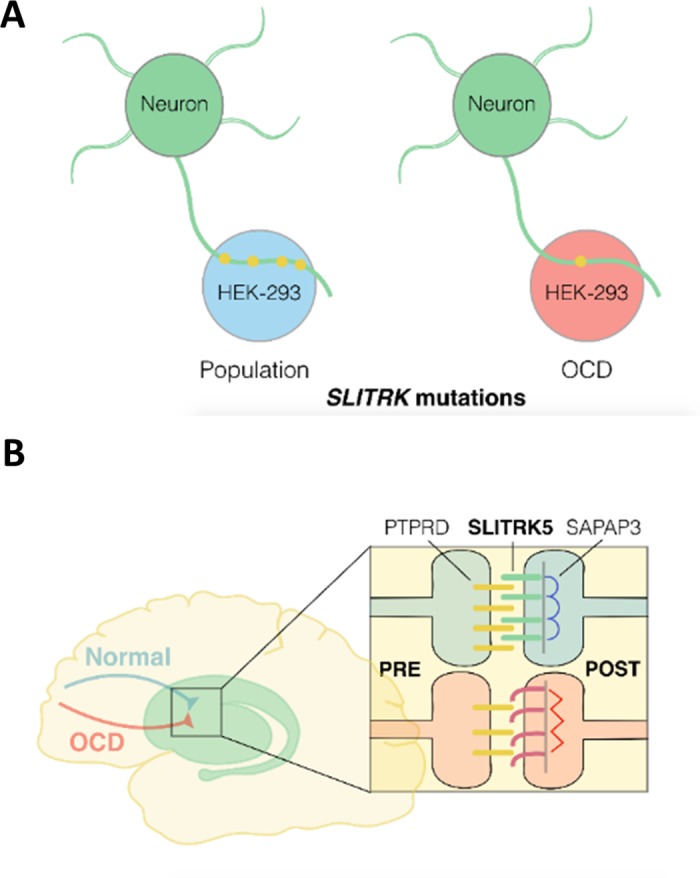
Genetic risk factors in OCD converge on corticostriatal synapse function. (A) Schematic of functional effects of OCD versus 1000 Genomes population control RNM’s. All control RNM’s supported synaptogenesis similar to WT Slitrk5 when expressed in HEK-293 cells (blue) while all OCD RNM’s impaired synaptogenesis (red). (B) Schematic of corticostriatal synaptic dysfunction identified in genetic studies of OCD. *PTPRD* is associated with OCD in GWAS and both *SLITRK5* and *SAPAP3* cause an OCD-like syndrome in knockout mice and contain rare OCD-associated mutations.

Impaired synaptic function in corticostriatal circuitry is an emerging theme in the genetics of OCD [31, 32]. Impaired synaptogenesis, either through decreased surface expression or PTPδ binding, is a shared cellular phenotype of the OCD RNM’s we identified (Fig 5). The SAPAP3 knockout mouse was the first model of serotonin reuptake inhibitor-sensitive OCD-like overgrooming phenotype [33]. SAPAP3, like SLITRK5, is highly enriched at excitatory post-synaptic densities in striatal neurons where it acts as a scaffolding protein [33]. Also similar to SLITRK5 knockout mice, SAPAP3 knockouts have reduced post-synaptic responses to stimulation of cortical input through altered expression of glutamate receptor subunits [34, 35]. Extending the genetic evidence for a role of altered corticostriatal synapse function in OCD, a polymorphism upstream of the human PTPδ gene (*PTPRD*) was the most significantly associated polymorphism in a recent large-scale GWAS of OCD [5] suggesting that common genetic effects on PTPδ-SLITRK5 interactions could contribute to risk for OCD in addition to the effects of the rare *SLITRK5* mutations we have identified here (Fig 5B).

## Supporting Information

S1 Fig TrkB binding is intact in most of OCD-associated SLITRK5 mutations. (A) Representative blots showing co-immunoprecipitation of TrkB to Slitrk5 alleles. Three mutations (N99K, E600K, and G722Δ) from OCD subjects displayed intact co-precipitation with TrkB whereas the A851V mutation exhibited complete loss of TrkB binding. (B) Densitometric quantification of the results shown on (A). Results are means ± SEM from 3 independent experiments. ****P<0.0001 compared to WT.
(DOCX)
