# Supplementary material for: Rare Synaptogenesis-Impairing Mutations in SLITRK5 Are Associated with Obsessive Compulsive Disorder
Source: PLoS One. 2017 Jan 13;12(1):e0169994. doi: 10.1371/journal.pone.0169994 (PMC5234816; doi:10.1371/journal.pone.0169994)
Supplement: S1 Fig — (A) Representative blots showing co-immunoprecipitation of TrkB to Slitrk5 alleles. Three mutations (N99K, E600K, and G722Δ) from OCD subjects displayed intact co-precipitation with TrkB whereas the A851V mutation exhibited complete loss of TrkB binding. (B) Densitometric quantification of the results shown on (A). Results are means ± SEM from 3 independent experiments. ****P<0.0001 compared to WT. (DOCX) [file pone.0169994.s001.docx]

**Supplementary Figures and Methods**

**Rare Synaptogenesis-Impairing Mutations in *SLITRK5* are Associated with Obsessive Compulsive Disorder**

Minseok Song, Carol A. Mathews, S. Evelyn Stewart, Sergey V. Shmelkov, Jason G. Mezey, Juan L. Rodriguez-Flores, Steven A. Rasmussen, Jennifer C. Britton, Yong-Seok Oh, John T. Walkup, Francis S. Lee, Charles E. Glatt

**Supplementary Figure S1**

**Supplementary Materials and Methods**


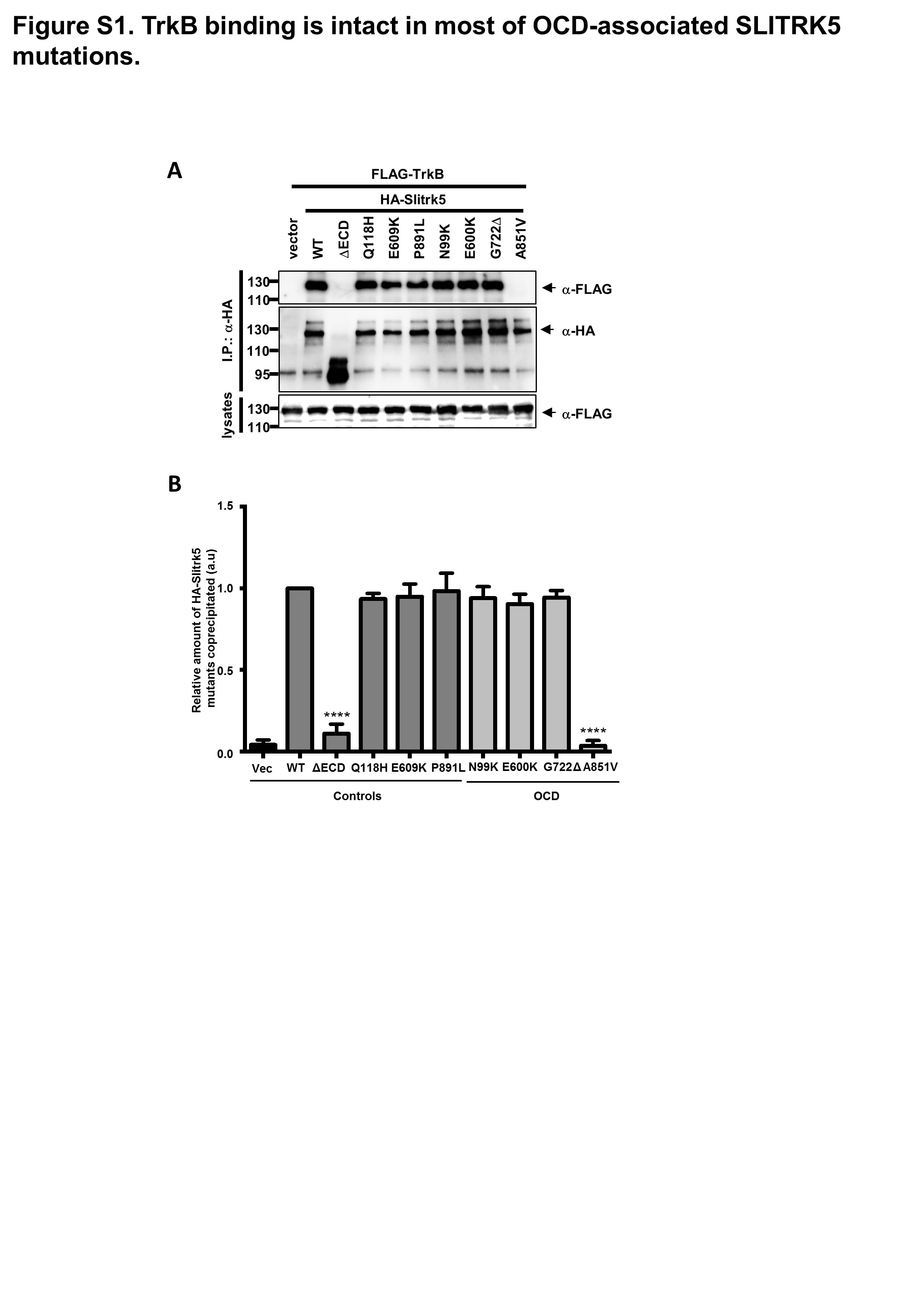


Figure S1. TrkB binding is intact in most of OCD-associated SLITRK5 mutations. (A) Representative blots showing co-immunoprecipitation of TrkB to Slitrk5 alleles. Three mutations (N99K, E600K, and G722Δ) from OCD subjects displayed intact co-precipitation with TrkB whereas the A851V mutation exhibited complete loss of TrkB binding. (B) Densitometric quantification of the results shown on (A). Results are means ± SEM from 3 independent experiments. ****P<0.0001 compared to WT.

**Material and Methods**

**Sequencing**

Forward and reverse primers were developed to amplify the full protein-coding sequence of *SLITRK5:*

| SLITRK5_a_fwd | AGGAGCAGCATATTTGTGGTGT |
| --- | --- |
| SLITRK5_a_rev | AGAGACGGTTCAAAAGGTTTCC |
| SLITRK5_b_fwd | TTATGGGGAAATCTGTGACAATG |
| SLITRK5_b_rev | GCTCACAAGAACAATTCCAAGG |
| SLITRK5_c_fwd | ACCCAACAATCTTTTCCGTTTT |
| SLITRK5_c_rev | AGAGATCTGCAGGTTGCAAGAG |
| SLITRK5_d_fwd | GTTTACAAACCCCCTTTGAAGC |
| SLITRK5_d_rev | CCTGCAGGAGGTTGTTATTCAA |
| SLITRK5_e_fwd | CTCTACCTGAATGGCAACAGGA |
| SLITRK5_e_rev | GACCTGGATAGAGGAGGGTGTG |
| SLITRK5_f_fwd | CCTAGTGGACGAGGTGATCTGT |
| SLITRK5_f_rev | CGTGCAGGTCTTTGTAATCCTC |
| SLITRK5_h_fwd | CTGCCCAAGGTGAAGACG |
| SLITRK5_h_rev | CGGTTGGGTTCTACAAAGACAG |
| SLITRK5_i_fwd | TTACAGGGGCATTTTAGAACCA |
| SLITRK5_i_rev | CTTAAAGCCCTTCTCCACACCT |
| SLITRK5_j_fwd | TTCTCAGCTTCGGTGGAAGATA |
| SLITRK5_j_rev | ACACCTCTGCAAAATCATAGGC |
| SLITRK5_k_fwd | CCTCCCTTCTCATTCCTTTTCT |
| SLITRK5_k_rev | AAACAATCGAAAATCGGCTACA |
| SLITRK5_l_fwd | CAGAGCTGATTGTATCCCAATG |
| SLITRK5__rev | TTTTGCTAATTAGGGGAAACACA |

All fragments were amplified in 20µl reactions consisting of: 25ng total genomic DNA, 10 pmol of each primer, 10µl New England Biolabs 2xPCR master mix.

Amplified fragments were treated with Exonuclease I (2U) and Shrimp Alkaline Phosphatase (2U) at 37^O^ C for one hr and inactivated at 80°C for twenty min.

PCR products were then sequenced by standard Sanger sequencing and mutations identified by visual inspection. All samples, which displayed putative mutations on sequencing, were re-sequenced on the reverse strand to confirm the presence of a mutation.

**Functional Assays**

**Reagents and Antibodies**

Mouse anti-MAP2 antibody (MAB3420) was purchased from Millipore (Temecula, CA, USA). Mouse anti-HA antibody (HA.11) was purchased from Covance (Emeryville, CA, USA). Goat anti-hemagglutinin (HA) (ab9134) was obtained from Abcam (Cambridge, MA, USA). Alexa Fluor dye-conjugated secondary antibodies and all cell culture reagents were obtained from Invitrogen (Carlsbad, CA, USA). The other reagents were from Sigma-Aldrich.

**Plasmid Constructs**

Human Slitrk5 cDNA (OpenBiosystems, MGC Human SLITRK5 Sequence-Verified cDNA, Accession: BC098106, Clone ID: 40012375) was subcloned into pCCL-PGK vector by using BamHI and XhoI sites. The amino terminal HA epitope tag was added to the 5′ end of Slitrk5 by PCR. HA-ΔECD Slitrk5 and all the control or OCD-specific Slitrk5 variants were generated by a PCR based methods. Extracellular domain of human PTPδ (OpenBiosystems, MGC Human PTPδ Sequence-Verified cDNA, Accession: BC106714, Clone ID: 40027582) was subcloned into pSecTag2 vector by using HindIII and XhoI sites to generate Fc-tagged version of PTPδ ECD. All of the constructs were confirmed by DNA sequence to exclude potential PCR-introduced mutations.

**Cell culture**

All reagents used to prepare primary neuronal cultures were purchased from Invitrogen, except glucose that was from Sigma. For neuronal cultures, the hippocampus of E16 mouse embryos were dissected in Ca^2+^- and Mg^2+^-free Hanks’ balanced salt solution supplemented with 0.37% glucose and digested in the same medium supplemented with 0.05% trypsin. Hippocampal cells were mechanically dissociated with fire-polished Pasteur pipettes and plated in plating medium (MEM containing 2 mM glutamine, supplemented with 10% FBS, 1 mM pyruvate, 0.37% glucose, and 25 U/ml penicillin/streptomycin). Hippocampal neurons were grown on poly-D lysine/laminin-coated glass coverslips at a cell density of 1.5x10^4^ cells/cm^2^ for immunocytochemistry. Plated neurons were kept in a humidified incubator at 37°C and 5% CO_2_. Cells were maintained in serum-free Neurobasal medium with B-27 supplement, 0.5 mM glutamine, 25 μM glutamate, and 25 U/ml penicillin/streptomycin, and AraC (Cytosine-1-β-D-arabinofuranoside) was added at DIV2. Cultures were grown for 6 days before being used for experiments, and media were changed every 3 days. COS7 cells were maintained at 37°C and 5% CO_2_ in Dulbecco’s Modified Eagle's medium (DMEM) with 1 mM Pyruvate, 10% fetal bovine serum (FBS), and 25 U/ml penicillin/streptomycin. Cells were transfected with Lipofectamine 2000 transfection reagent following the manufacturer’s instructions (Invitrogen). 48 hr after transfection, experiments were performed.

**Western blot analysis and Immunoprecipitation**

Cell line cultures were lysed in RIPA buffer (150 mM NaCl, 20 mM Tris pH 8.0, 1 mM EDTA, 1% Triton X-100, 0.5% DOC, 0.1% SDS) containing protease and phosphatase inhibitors (2 μg/ml leupeptin, 2 μg/ml aprotinin, 1 mM sodium orthovanadate, 10 mM sodium fluoride, and 1 mM phenylmethylsulfonyl fluoride). Lysates were clarified by centrifugation at 12,000 g for 15 min and protein concentrations were determined by Lowry assays (DC protein assay kit, Bio-Rad). The supernatant was incubated with antibody-coupled agarose beads for overnight at 4°C, with constant rotation. After washing three times with the lysis buffer, the bound proteins were eluted by boiling in LDS sample buffer for 5 min. Eluted proteins were separated on a 10% Nupage Bis-Tris Gel (NuPAGE® Novex, Invitrogen, Carlsbad, CA) and transferred to PVDF membranes. These were then blocked for 1 h in TBS with 0.1% Tween 20 (TBS-T) and 5% low-fat milk. The incubation with the primary antibodies was performed overnight at 4ºC in TBS-T with 3% BSA, followed by washes in TBS-T and incubation with HRP secondary antibodies at room temperature for 1 h. Immunoreactive proteins were visualized by ECL detection and film autoradiography. Striping was performed by washing the membranes in 0.1M Glycine pH 2.5 for 15 min, followed by another wash in 1% SDS for 15 min. Each experiment was repeated at least three times.

**Subcellular localization assay**

To quantitatively analysis subcellular localization of Slitrk5 variants, COS7 cells were transfected with HA-tagged Slitrk5 variants. Forty-eight hr after transfection, COS7 cells were fixed and incubated with anti-HA antibodies (HA.11) without permeabilization to specifically label cell surface HA-tagged Slitrk5 variants. Surface HA-tagged Slitrk5 variants were visualized with Alexa-488 dye-conjugated anti-mouse antibodies (dilution 1/300) for 20 min at room temperature.. Then, cells were permeabilized with 0.2% Triton X-100-containing PBS, and internalized Slitrk5 variants were stained with anti-HA antibodies (HA.11). Internally localized HA-Slitrk5 variants were visualized with Alexa-568 dye-conjugated anti-mouse antibodies (dilution 1/300) at room temperature for 20 min. Cells were examined by fluorescence microscopy and staining intensities of each fluor in individual cells were quantified using NIS-Elements (Nikon Instruments Inc., NY, USA). Values corresponding to the surface Slitrk5 (green) were divided by the total fluorescence values (red + green) and normalized to untreated controls. Each experiment was repeated at least three times.

**Production of soluble PTPδ-Fc protein and binding assays**

Based on previously described methods[^14^](#_ENREF_14), soluble PTPδ ectodomain fused to Fc (PTPδ-Fc) were generated using HEK-293 cells transfected with the expression vectors and purified from culture media. For testing binding of soluble PTPδ-Fc, HEK-293 cells on coverslips were transfected with the expression vectors for WT and variants of HA-tagged Slitrk5 and grown for 48 hr. The transfected cells were washed with extracellular solution (ECS; containing, in mM: 168 NaCl, 2.4 KCl, 20 HEPES pH 7.4, 10 D-glucose, 2 CaCl_2_, 1.3 MgCl_2_) that contained 100 μg/ml BSA (ECS/BSA) and then incubated with ECS/BSA that contained 200 nM purified PTPδ Fc-fusion protein for 1 h at room temperature. The cells were washed in ECS, fixed with 4% paraformaldehyde, incubated with blocking solution, and then with mouse anti-HA antibody. Cells were incubated with subtype-specific fluorescenated secondary antibodies and analyzed by fluorescence microscopy with Alexa488 dye–conjugated anti-human IgG (H+L) antibodies (donkey IgG; 1:300; Jackson ImmunoResearch) for labeling of bound Fc proteins. For quantification, we measured the average intensity of bound Fc protein per HEK-293 cell area, subtracted for off-cell background.

**Synapse formation assay with mixed-cell culture**

HEK-293 cells were transfected with WT and variants of Slitrk5 constructs. 24 hr after transfection, at days 6-7 in vitro of the hippocampal neuron culture, HEK-293 cells expressing various Slitrk5 constructs were detached with trypsin and dissociated. Trypsin-containing solutions were removed by centrifugation, and dissociated HEK-293 cells were seeded atop the hippocampal neurons grown on glass coverslips. 24 hr later, mixed culture of neurons and HEK-293 cells were fixed with a 4% paraformaldehyde solution in PBS for 15 min at room temperature and permeablized with 0.2% Triton X-100 in PBS for 5 min. After washing neurons with PBS three times, they were blocked with 10% donkey serum, 3% bovine serum albumin in PBS for 30 min. Specimens were incubated with primary antibodies and then incubated with subtype-specific fluorescenated secondary antibodies. Coverslips were mounted with prolong gold antifade reagent (Invitrogen, CA).

For image acquisition and quantification, the total intensity of synapsin I signal in regions positive for both surface HA (labeling transfected HEK-93 cells) and dephospho-tau (labeling axons) was measured. Analysis was performed using NIS-Elements, Microsoft Excel and GraphPad Prism 4. Statistical comparisons were made using one-way ANOVA with Dunnett's multiple comparisons test, as indicated in the figure legends. All data are reported as the mean ± s.e.m. from at least two, mostly three, independent experiments and statistical significance was defined as *P< 0.05, **P< 0.01, ***P< 0.001, ***P< 0.0001.
